# Validity of the Self-administered Food Frequency Questionnaire Used in the 5-year Follow-up Survey for the JPHC Study to Assess Folate, Vitamin B_6_ and B_12_ Intake: Comparison with Dietary Records and Blood Level

**DOI:** 10.2188/jea.13.1sup_98

**Published:** 2007-11-30

**Authors:** Hiroyasu Iso, Yuri Moriyama, Kaori Yoshino, Satoshi Sasaki, Junko Ishihara, Shoichiro Tsugane

**Affiliations:** 1 Department of Public Health Medicine, Institute of Community Medicine, University of Tsukuba.; 2 The Public Health Institute of Kochi Prefecture.; 3 Epidemiology and Biostatistics Division, National Cancer Center Research Institute East.; 4 Cancer Information and Epidemiology Division, National Cancer Center Research Institute.

**Keywords:** validity, questionnaire, folate, vitamin B_6_, vitamin B_12_

## Abstract

To validate a self-administered food frequency questionnaire (FFQ) for the estimation of dietary intake of folate, vitamins B_6_ and B_12_, we compared measures of these vitamins by the FFQ and plasma concentrations among 87 male volunteers aged 40-69 years in the Japan Public Health Center-based prospective study (JPHC Study). No men used folate, vitamins B_6_ or B_12_ as supplements. There was a moderate association between energy-adjusted dietary intake and plasma concentration for folate and vitamin B_6_. The Spearman correlation coefficient was 0.26 and 0.23, respectively, in the total samples. These correlation coefficients were slightly higher when men in the Ishikawa PHC area were excluded from the analysis; the correlation coefficient was 0.29 for folate and 0.26 for vitamin B_6_. No significant correlation was found between dietary vitamin B_12_ intake and serum B_12_ concentration; the correlation coefficient was 0.06 in the total sample and 0.15 when the Ishikawa men were excluded from the analysis. In conclusion, we found that the FFQ used for the 5-year follow-up survey of JPHC Study could reasonably rank individuals by dietary intake of folate and vitamin B_6_, but not vitamin B_12_, among Japanese community-dwelling population samples.

Dietary intake of folate, vitamins B_6_ and B_12_ has been associated with blood concentrations of homocysteine, which was found to be a risk factor for coronary heart disease and ischemic stroke.^1^ Some studies also suggested that the intake of these vitamins reduces the risk of cardiovascular disease, through an independent effect of blood homocysteine concentrations.^2^^,^^3^ However, these data were mostly from case-control studies, and the information from prospective studies was limited to Caucasian populations. A prospective study is warranted to examine the relation of such vitamin intake and the risk of cardiovascular disease among Japanese, whose dietary habits are markedly different from Caucasians. The Japan Public Health Center-based prospective Study on cancer and cardiovascular diseases (JPHC Study)^4^ is an attempt to examine the relations between various health behaviors and the risk of cancer and cardiovascular disease. In the present study, we examined the validation of a self-administered food frequency questionnaire (FFQ) by comparing measures of folate, vitamins B_6_ and B_12_ intake by FFQ and plasma concentrations in a community-dwelling population of Japanese men.

## METHODS

The subjects of the present validation study were a subsample of the participants in the JPHC Study Cohort I, aged 40 to 69 years old in four Public Health Center (PHC) areas: Ninohe, Yokote, Saku and Ishikawa.^5^ An FFQ with 138 food items was filled out by subjects from the Ninohe, Yokote and Saku PHC areas in February of 1995, and by subjects from the Ishikawa PHC area in February of 1996. Blood samples were collected in February (winter) and August (summer) of 1994 from the subjects of the Ninohe, Yokote and Saku PHC areas, and in February (winter) and August (summer) of 1995 from the subjects in the Ishikawa PHC area. In this study, we included 87 men (19 to 25 men from each PHC) with their plasma folate, vitamins B_6_ and B_12_ levels.

Dietary intakes of folate, vitamins B_6_ and B_12_ were estimated using the FFQ. Standard Tables of Food Composition in Japan (5th revised edition for new food items)^6^ and the United States Department of Agriculture (USDA) Nutrient Database for Standard Reference^7^ were used to calculate the intakes. Contents of folate in rice and seaweed were analyzed at the Japan Food Analysis Center (Tokyo) because these values were not available in either the Japan Tables or the USDA Nutrient Database.

Blood was drawn by a 10-ml heparinized tube, and the plasma was separated. A 1-ml aliquot was stored at -80°C until the analysis. Plasma total homocysteine was measured by high-performance liquid chromotograhy. Plasma folate and vitamin B_12_ were measured by chemiluminescence immunoassay using the kits from Chiba-Corning (Medfield, MA). Vitamin B_6_ was assayed by high-performance liquid chromatography as pyridoxal-5'-phosphate (PLP) using a kit from Immundiagnostik (Germany).

For statistical analyses, we compared the measures of folate, vitamin B_6_ and B_12_ by the food frequency questionnaire with those by mean plasma concentrations obtained in winter and summer. Mean dietary intake was presented as both crude mean and mean value per 1000 kcal of energy, while mean plasma concentrations were shown for each PHC area. Energy-adjusted dietary intake according to the residual model^8^ was used to calculate the Spearman correlation coefficients between these vitamin intakes and average plasma concentrations in winter and summer. To examine the validity of quintile classification according to the FFQ, mean plasma concentrations were calculated in each quintile subgroup and ratio of concentrations in the lowest vs. higher quintiles. Furthermore, the quintiles of dietary intakes and plasma concentrations were cross-tabulated to compare these two measures.

## RESULTS

Dietary intakes of folate, vitamin B_6_ and B_12_ in the four surveyed areas were shown in Table 1. Mean crude folate intake was lowest in Ishikawa, intermediate in Ninohe and Yokote areas, and highest in the Saku area. Mean folate intake per 1,000 kcal of energy, however, was lower in Ninohe than in the other three areas. Both mean values of crude vitamin B_6_ intake and vitamin B_6_ intake per 1,000 kcal of energy were lower in Ishikawa than in the other three areas. Both mean values of crude vitamin B_12_ intake and vitamin B_12_ intake per 1,000 kcal of energy were lowest in Ishikawa, intermediate in Yokote and Saku, and highest in Ninohe.

**Table 1.** Dietary intake of folate, vitamin B_6_, and B_12_ assessed with FFQ by area in men

	Ninohe PHC area		Yokote PHC area		Saku PHC area		Ishikawa PHC area		ANOVA

	Mean ± SD	Median	Mean ± SD	Median	Mean ± SD	Median	Mean ± SD	Median	p-value
Men	n = 22		n = 25		n = 19		n = 21		
Crude values									
Folate (*μ*g/day)	332 ± 124	330	307 ± 95	287	392 ± 153	380	260 ± 131	225	0.012
Vitamin B_6_ (mg/day)	2.47 ± 0.94	2.2	2.07 ± 0.9	1.83	2.23 ± 0.69	2.09	1.42 ± 0.59	1.33	0.000
Vitamin B_12_ (*μ*g/day)	18.9 ± 12.5	15.8	12 ± 6.41	8.76	13.2 ± 6.86	11.8	6.95 ± 4.43	6.12	0.000
Energy-adjusted values									
Folate (*μ*g/day)	117 ± 26.4	113	136 ± 32.3	142	148 ± 35	145	145 ± 41.1	137	0.017
Vitamin B_6_ (mg/day)	0.86 ± 0.17	0.83	0.89 ± 0.13	0.88	0.85 ± 0.11	0.82	0.80 ± 0.13	0.80	0.171
Vitamin B_12_ (*μ*g/day)	6.4 ± 3.2	6.5	5.2 ± 2.3	4.3	4.9 ± 1.7	4.8	4 ± 2.5	3.7	0.017

Table 2 shows the plasma concentrations of folate, vitamins B_6_ and B_12_ in winter and summer in the four surveyed areas. Mean plasma folate was lower in winter than in summer in Ishikawa, but the opposite trend was observed for the other areas. Mean vitamin B_6_ was lower in winter than in summer in all but the Ishikawa area, where no seasonal variation was observed. Mean vitamin B_12_ was lower in summer than in winter in all four areas. The mean concentrations of these vitamins in the two seasons were calculated for comparison of plasma vitamin levels among the four areas. Mean plasma folate was lower in Ninohe than in the other three areas. Mean plasma vitamin B_6_ was lower in Saku than in the other areas. Mean plasma vitamin B_12_ was lowest in Yokote, intermediate in Saku and Ishikawa and highest in Ninohe.

**Table 2.** Plasma concentrations of folate, vitamins B_6_ and B_12_ by area and season in men

	Ninohe PHC area		Yokote PHC area		Saku PHC area		Ishikawa PHC area		ANOVA

	Mean ± SD	Median	Mean ± SD	Median	Mean ± SD	Median	Mean ± SD	Median	p-value
Winter									
Folate (nmol/L)	13.9 ± 5.0	12.7	19.1 ± 6.9	19.0	18.8 ± 7.4	17.0	15.3 ± 5.2	14.8	0.015
Vitamin B_6_ (nmol/L)	101 ± 94	82	93 ± 54	84	75 ± 41	77	126 ± 82	153	0.388
Vitamin B_12_ (pmol/L）	538 ± 225	431	425 ± 118	452	592 ± 222	554	494 ± 195	466	0.035
Summer									
Folate (nmol/L)	13.2 ± 4.8	12.2	17.7 ± 7.4	17.0	15.1 ± 5.8	13.4	24.1 ± 13.0	19.5	0.000
Vitamin B_6_ (nmol/L)	142 ± 81	148	147 ± 103	117	80 ± 49	72	123 ± 131	86	0.237
Vitamin B_12_ (pmol/L)	376 ± 234	305	312 ± 144	265	355 ± 157	325	384 ± 152	347	0.498
Average of winter and summer									
Folate (nmol/L）	13.6 ± 4.5	12.5	18.4 ± 6.4	19.5	16.9 ± 4.8	15.7	18.8 ± 7.3	15.2	0.017
Vitamin B_6_ (nmol/L)	121 ± 114	79	120 ± 76	103	77 ± 44	73	125 ± 122	89	0.351
Vitamin B_12_ (pmol/L)	457 ± 208	377	368 ± 112	360	473 ± 169	461	443 ± 151	411	0.135

Table 3 presents the correlation coefficients between dietary folate, vitamin B_6_ and B_12_ and the plasma concentrations of these vitamins. The correlation coefficients between crude dietary intake and average plasma concentrations of folate and vitamin B_6_ in total samples were 0.05 and 0.17, respectively. The corresponding correlation coefficients after the dietary intakes were adjusted for energy were 0.26 and 0.23, respectively. These correlation coefficients were slightly higher when the Ishikawa samples were excluded from the analysis, i.e., correlation coefficients of 0.29 and 0.26, respectively (data not shown). The correlation coefficients between dietary intake and plasma concentration of vitamin B_12_ were 0.001 for crude intake and 0.06 for energy-adjusted intake. A steady increase in mean intake from the lowest to the highest quintile was observed only for folate (Table 4). One-third of the men had the same category of the quintiles of dietary intake plasma concentrations for folate (Table 5). The respective proportions were 20% for vitamin B_6_, against only 11% for vitamin B_12_.

**Table 3.** Crude values of dietary folate (*μ*g/day), vitamins B_6_ (mg/day) and B_12_ (*μ*g/day) assessed with FFQ, and plasma concentrations of folate (nmol/L), vitaminB_6_ (nmol/L), and B_12_ (pmol/L) and their correlation coefficients in men (n=87)

	FFQ^1^			NPlasma			Spearman correlation	
		
	Mean ± SD	Median	Range	Mean ± SD	Median	Range	Crude	Energy-adjusted^2^
Folate	321 ± 131	290	111-856	16.9 ± 6.1	15	5.8-33.1	0.05	0.26
Vitamin B_6_	2.05 ± 0.88	1.87	0.76-5.71	112 ± 95	83	28-532	0.17	0.23
Vitamin B_12_	12.8 ± 9.1	9.9	1.4-46.5	432 ± 165	392	143-873	0.001	0.06

^1^FFQ, food frequency questionnaire.

^2^Energy was adjusted by residual model for intake.

For n=87, r>0.21 = p<0.05, r>0.28 = p<0.01, r>0.35 = p<0.001.

**Table 4.** Crude values of dietary folate, vitamins B_6_ and B_12_ assessed with FFQ, according to quintile of plasma concentrations in men

Dietary intake	Quintile of plasma concentration																							

	Lowest				2nd					3rd					4th					ighest				
				
	(n)	Mean	±	SD	(n)	Mean	±	SD	Ratio	(n)	Mean	±	SD	Ratio	(n)	Mean	±	SD	Ratio	(n)	Mean	±	SD	Ratio
Folate (*μ*g/day)	(18)	297	±	86	(17)	304	±	72	1.02	(18)	317	±	60	1.07	(17)	322	±	89	1.08	(17)	361	±	107	1.22
Vitamin B_6_ (mg/day)	(17)	1.97	±	0.27	(18)	1.93	±	0.22	0.98	(17)	2.02	±	0.30	1.03	(18)	2.03	±	0.37	1.03	(17)	2.10	±	0.28	1.07
Vitamin B_12_ (*μ*g/day)	(17)	13.5	±	8.3	(17)	10.7	±	6.1	0.79	(18)	12.5	±	5.7	0.93	(17)	11.5	±	3.9	0.85	(17)	12.4	±	5.0	0.92

**Table 5.** Comparison of FFQ with plasma concentrations for folate, vitamins B_6_ and B_12_ based on joint classification by quintile (%) in men.

	Same category	Adjacent category	Extreme category
Folate	31	57	5
Vitamin B_6_	20	55	5
Vitamin B_12_	11	41	4

## DISCUSSION

We found a moderate association between dietary intake and plasma concentrations of folate and vitamin B_6_ when the intakes were estimated by the FFQ used for the 5-year follow-up survey. However, the estimation of dietary intake of vitamin B_12_ was not associated with plasma concentrations of vitamin B_12_. This lack of correlation may be in part due to the larger inter-individual variability in the absorption of vitamin B_12_ compared with that of folate and vitamin B_6_.^9^

The validity of the food frequency questionnaire used in the Nurses' Health Study was tested among Caucasian samples of 57 men and 82 women for the estimation of dietary intake of folate, vitamins B_6_ and B_12_.^10^ The correlation coefficients between dietary intake and plasma concentration were 0.62 for folate, -0.16 for vitamin B_6_, and 0.34 for vitamin B_12_. When persons using these vitamin supplements were excluded from the analysis, the respective correlation coefficients were 0.51, 0.04, and 0.20. Compared with these correlation estimates, ours were somewhat lower for folate and vitamin B_12_ and higher for vitamin B_6_. In the Framingham Heart Study in which the same questionnaire as in the Nurses' Health Study was used, the correlation between dietary intake and plasma concentration was found to be moderate for folate and vitamin B_6_, but low for vitamin B_12_.^9^ Therefore, the dietary measure for folate was consistently valid in two Caucasian studies. In the present study, the correlation was higher for folate and vitamins B_6_ than for B_12_. The population level of dietary folate intake was correlated with the population level of plasma folate level; the Ishikawa men showed a lower dietary intake and plasma folate level than men in the other three PHC areas.

The validity of the dietary measures for vitamin B_6_ and B_12_ has not been consistent among various studies. The dietary and biochemical measures of vitamin B_6_, for example, were moderately associated in the Framingham Heart Study and our study, but not in the Nurses' Health Study. The measures for vitamin B_12_ were moderately associated in the samples of the Nurses' Health Study, but not in the Framingham Heart Study or the present study.

Low crude folate intake was observed in Ishikawa. Since a low dietary folate intake is a strong determinant for high plasma homocysteine concentrations which may increase the risk of cardiovascular disease,^1^^,^^2^ we will examine the adverse health effect in Ishikawa men in the PHC cohort analyses.

In conclusion, we found that the FFQ used for the 5-year follow-up survey of JPHC Study could reasonably rank individuals by dietary intake of folate and vitamin B_6_ for epidemiological use among Japanese community-dwelling population samples. The estimate of vitamin B_12_ from the FFQ, however, might not be reliable.
